# Clinical insight in schizophrenia: Time-specific associations with symptoms, cognition and antipsychotic mechanisms

**DOI:** 10.1016/j.scog.2026.100463

**Published:** 2026-09-17

**Authors:** Lena Antonsen Stabell, Erik Johnsen, Rune Andreas Kroken, Else-Marie Løberg, Inge Joa, Solveig Klæbo Reitan, Maria Rettenbacker, Rolf Gjestad

**Affiliations:** a Division of Psychiatry, Haukeland University Hospital, Bergen, Norway; b Department of Clinical Medicine, University of Bergen, Bergen, Norway; c Mohn Research Centre for Psychotic Disorders, Bergen, Norway; d Faculty of Psychology, Department of Clinical Psychology, University of Bergen, Bergen, Norway; e TIPS, Centre for Clinical Research in Psychosis, Stavanger University Hospital, Stavanger, Norway; f Institute of Public Health, Faculty of Health Sciences, University of Stavanger, Stavanger, Norway; g Department of Mental Health, Nidelv DPS, St. Olav University Hospital, Trondheim, Norway; h Department of Mental Health, Faculty of Medicine and Health Sciences, NTNU, Trondheim, Norway; i Department of Psychiatry, Psychotherapy and Psychosomatics, Medical University of Innsbruck, Innrain 52, Innsbruck, Austria; j Centre for Research and Education in Forensic Psychiatry, Haukeland University Hospital, Bergen, Norway

**Keywords:** Awareness, Schizophrenia Spectrum disorders, Cognitive functioning, Dopaminergic mechanisms, Dose-response relationship

## Abstract

**Background:**

Impaired clinical insight in schizophrenia is associated with poor treatment adherence and outcomes. Previous research on the influence of symptoms, cognition, and antipsychotic exposure has been inconclusive and restricted to multi-episode samples. We examined time-specific associations between overall psychopathology, cognitive functioning, antipsychotic D₂ receptor mechanisms, antipsychotic exposure and dosage, and insight, whether these associations changed over time, and whether dosage moderated them.

**Methods:**

In 144 individuals with schizophrenia, including antipsychotic-naïve and previously treated participants, multiple regressions were conducted at five cross-sectional time points and examined changes in associations over one year. Insight was assessed using PANSS item G12. Symptom severity was represented by the sum of the remaining PANSS items (mPANSS). Antipsychotic exposure was quantified as cumulative Defined Daily Doses and classified as dopamine antagonists (DA) or partial agonists (DPA). Cognitive functioning was expressed as a mean t-score.

**Results:**

Greater symptom severity was consistently associated with poorer insight across time points and was the only variable showing a linear increase in strength over time. Higher DPA doses were associated with poorer insight at baseline and 12 months, but improved insight at 12 weeks. Higher cognitive functioning was associated with better insight at baseline and interacted with lower doses at 6 weeks and 12 months. No consistent associations were observed in antipsychotic-naïve participants.

**Conclusion:**

Clinical insight was most consistently associated with symptom severity, with an increasingly robust association over time, highlighting the need to assess insight across the symptom spectrum, including when symptoms are mild and independently of antipsychotic dosage.

## Highlights

• Symptom severity was most consistently associated with of clinical insight
• Symptom severity–insight relation strengthens over time during follow-up
• Cognition showed weak, time-specific associations to insight
• Antipsychotic dose affected insight only via interactions
• No consistent advantage of specific antipsychotic mechanisms

## Graphical abstract

**Figure:**
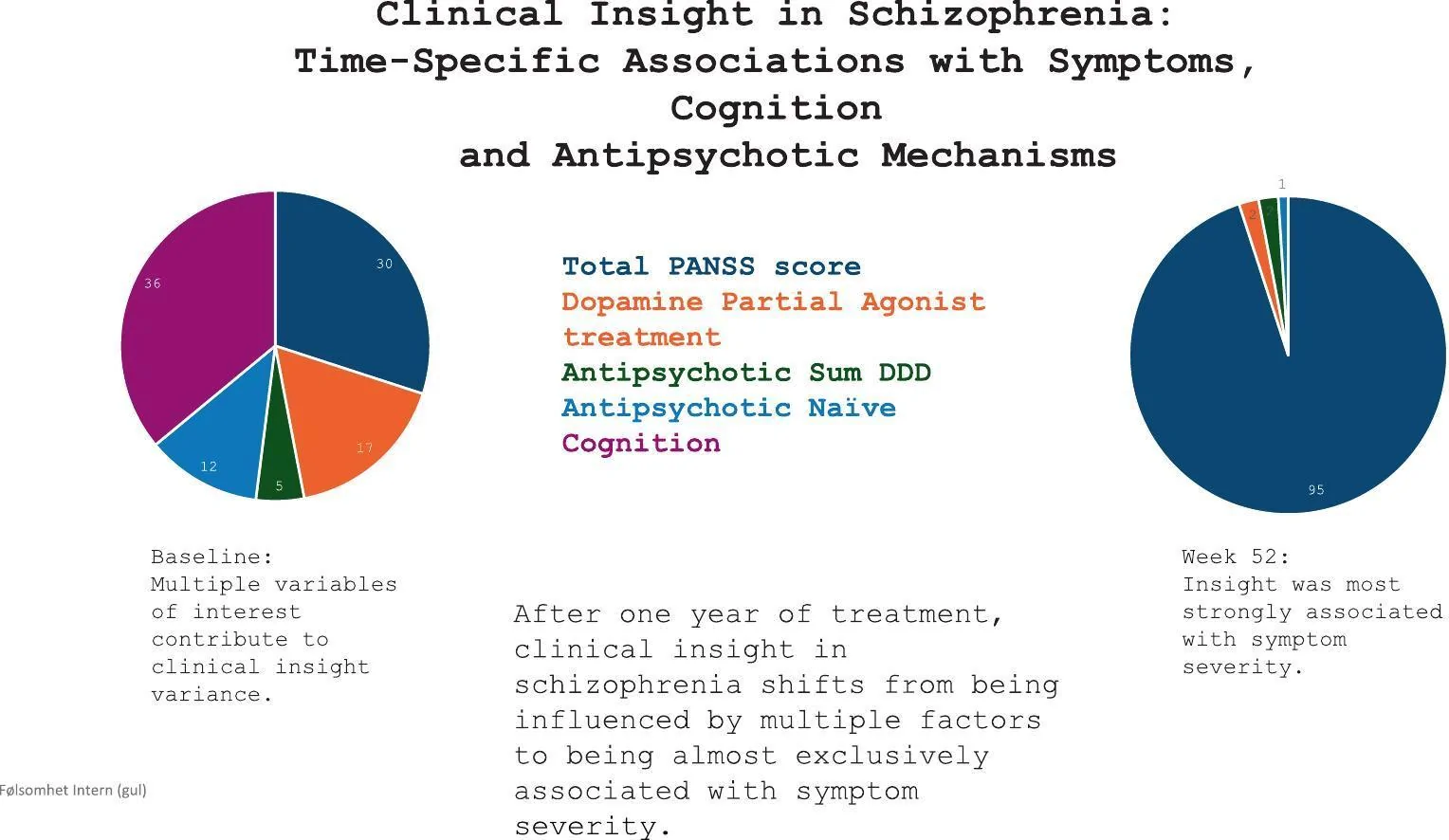

## 1 Introduction

Impaired clinical insight is commonly observed among patients with schizophrenia (Amador and Gorman, 1998; Lehrer and Lorenz, 2014). In the present study, the term clinical insight is used to refer to awareness of having a mental disorder, recognition of symptoms as part of that disorder, and awareness of the need for treatment (Amador and David, 2004; David, 1990). The term is used to distinguish these illness-related aspects of insight from broader insight constructs described in the literature and corresponds closely to the measure of insight used in the present study. Impaired clinical insight is associated with poorer treatment adherence, reported in both quantitative (Deng et al., 2022; Kim et al., 2020; Yaegashi et al., 2020) and qualitative studies (Clifford et al., 2020), and may affect outcome (Czobor et al., 2015; Lincoln et al., 2007) and increase hospitalisations (Zipursky et al., 2014). Differences in treatment history may influence clinical insight and medication response (Schennach et al., 2012; Seif et al., 2025).

Higher total scores on psychosis symptom scales, as well as elevated scores in specific symptom subcategories, are associated with impaired clinical insight (Lysaker et al., 2018). These include the positive, negative, and general psychopathology subscales from the Positive and Negative Syndrome Scale (PANSS), as well as dimensions derived from factor analyses of the same instrument (Monteiro et al., 2008; Stabell et al., 2026). A relationship between the reduction of psychosis symptoms and improved clinical insight has been shown (Bianchini et al., 2014; Mattila et al., 2017; Stabell et al., 2023). Insight has generally been associated with better clinical and functional outcomes. However, the relationship between insight and affective outcomes remains controversial, as greater insight has also been linked to depression, suicidality and poorer subjective wellbeing, a phenomenon referred to as the “insight paradox” (Lopez-Morinigo and David, 2024). Recent findings from the OPTiMiSE first-episode psychosis trial suggest that improvements in insight are associated with better clinical outcomes and do not appear to increase the risk of adverse outcomes (Lopez-Morinigo et al., 2026).

In addition to symptom severity and clinical outcomes, insight has been associated with cognitive functioning (Lysaker et al., 2018). While it seems intuitive to associate poor insight with cognitive deficits, both are complex constructs with both state and trait properties, which may explain the inconsistent findings in the literature. A meta-analysis published two decades ago identified several cognitive domains related to impaired insight (Aleman et al., 2006). These findings were later confirmed in a larger meta-analysis, which nevertheless found only small associations between insight and neurocognitive functioning (Nair et al., 2014). More recently, Subotnik et al. reported similarly weak associations, between insight and cognitive domains such as processing speed, attention/vigilance, working memory, verbal and visual learning and memory, and reasoning/problem solving (Subotnik et al., 2020).

Previous studies have investigated insight in relation to specific antipsychotic agents, including analyses from our own sample (Mohamed et al., 2009; Pijnenborg et al., 2015; Stabell et al., 2023). Although some studies have suggested differential effects of individual antipsychotics on insight, findings have not been consistent across samples. Furthermore, the relative importance of antipsychotic dose and broader pharmacological mechanisms remains uncertain. Antipsychotic medication primarily targets the dopamine system by blocking postsynaptic dopamine receptors and are therefore referred to as dopamine antagonists (DAs) (Cookson and Pimm, 2023; Grace and Uliana, 2023). However, the novel antipsychotics aripiprazole, brexpiprazole, and cariprazine, classified as dopamine partial agonists (DPAs), may enhance or preserve dopaminergic transmission in prefrontal cortical regions compared with traditional dopamine antagonists, thereby contributing to dopamine system stabilisation (Cookson and Pimm, 2023; Murphy et al., 2016). This is particularly relevant to impaired insight, as abnormalities in prefrontal cortex function have been associated with poorer clinical insight (Kim et al., 2023; Pijnenborg et al., 2020). Aripiprazole has indeed shown pro cognitive effects (Tamminga, 2002), but superiority over the classical DAs is not yet demonstrated (Anda et al., 2021).

Antipsychotic exposure can be measured in various ways, most commonly through D₂ receptor occupancy, Defined Daily Doses (DDD) or drug-specific equivalent doses (Lako et al., 2013). A D₂ receptor occupancy of around 80% is associated with maximum efficacy (Siafis et al., 2023), though lower doses often suffice for first-episode or antipsychotic-naïve individuals (Uchida et al., 2011). Occupancy levels above this threshold are generally associated with an increased risk of extrapyramidal side effects and cognitive impairment (Sakurai et al., 2013; Siafis et al., 2023), although dopamine partial agonists such as aripiprazole represent an important exception, achieving clinical efficacy at D₂ occupancies exceeding 90% while maintaining a relatively low propensity for motor side effects (Tuplin and Holahan, 2017; Yokoi et al., 2002). Clinicians must therefore balance dosage to optimise symptom reduction and insight while minimising risk of cognitive and other side effects.

Few studies have investigated the interplay between clinical insight, symptom severity, cognitive functioning, and antipsychotic exposure. One small dose-reduction study reported that both illness severity, operationalised as higher levels of psychotic symptoms, and cognitive functioning independently was associated with insight. Furthermore, these relationships were moderated by D₂ receptor occupancy: impaired insight was associated with cognitive dysfunction at higher occupancy levels, and with illness severity at lower levels (Gerretsen et al., 2017). The same variables were later investigated in the Clinical Antipsychotic Trials of Intervention Effectiveness (CATIE) study, in which illness severity emerged as the only independent association of insight, where while the other variables yielded no statistically significant main, moderation or mediation effects on clinical insight (Ozzoude et al., 2019). Thus, the contribution of cognitive functioning remains uncertain. This may be related to differences in study design and timing of assessments in the two studies, where the follow-up was at 28 weeks in the dose-reduction study and at 8 weeks in the CATIE study. Both study samples consisted of multi-episode patients, limiting the generalisability of findings to more heterogeneous clinical populations. Importantly, both studies examined two assessment points. Consequently, little is known about whether the within-time associations between insight and the variables of interests remain stable or vary across different stages of treatment.

Antipsychotic dosage influences both symptom severity and cognitive performance, with extensive D₂-receptor occupancy being associated with cognitive side effects that may, in turn, affect clinical insight. Notably, antipsychotic-naïve individuals often require lower doses to achieve symptom control and may therefore experience fewer cognitive impairments. These differences suggest that the interplay between cognition, symptoms, and insight may vary according to both the level and history of antipsychotic exposure. Drawing on previous research, we hypothesise that antipsychotic exposure, operationalised as cumulative dosage (Sum DDD) and prior treatment history, may moderate the relationship between symptom severity, cognitive functioning, and clinical insight.

Therefore, this study aimed to assess time-specific associations between insight and overall psychopathology, cognitive functioning, antipsychotic D₂ receptor mechanisms, prior antipsychotic exposure, and current antipsychotic dosage, examine whether these associations changed over time, and explore whether current dosage moderated the associations between psychopathology, cognitive functioning, and insight.

## 2 Methods

### 2.1 Design

The BeStInTro trial was a semi-randomised, longitudinal study, with a head-to-head comparison of amisulpride, aripiprazole, and olanzapine in patients with schizophrenia spectrum disorders (Johnsen et al., 2020). This paper reports on a secondary outcome of the trial. The pragmatic study design allowed participants to remain in the study even if they changed or discontinued the study drug. Consequently, the present analysis encompasses all antipsychotic treatments used during the study period, not only the three trial drugs.

### 2.2 Sample

Three study sites in Norway and one in Austria included patients from 20st of October 2011 to 30 December 2016. Last date of follow up was 21st of December 2017. Patients were required to be at least 18 years of age, have a diagnosis of schizophrenia spectrum disorder according to ICD-10 codes F20–F29 (World Health, 2004) and be in an active phase of psychosis, as indicated by a score of four or more on at least one of the following items of the Positive and Negative Syndrome Scale (PANSS) (Kay et al., 1987): P1 delusions, P3 hallucinations, P5 grandiosity, P6 suspiciousness/persecution or G9 unusual thought content. Patients were excluded from the study if they were pregnant or lactating, unable to understand the native language, presented organic psychosis, had hypersensitivity to the active substances in the study drugs, or somatic contraindications.

### 2.3 Measurements

All data was collected at baseline, 6 weeks, 3 months, 6 months, and 12 months, except the neurocognitive tests, which were not done at 3 months.

The Positive and Negative Syndrome Scale (PANSS) was used to assess psychosis symptoms, including Insight (item G12). Higher scores represent more symptoms and more impaired insight. All assessors were trained and certified by the PANSS Institute in New York. The PANSS has good reliability and validity (Kay et al., 1988). We use the term modified PANSS (mPANSS), which is PANSS total score minus item G12, when reporting on total psychosis symptoms.

Exposure to antipsychotics: We used DDD to quantify antipsychotic exposure. DDD is a unit indicating “the assumed average maintenance dose per day for a drug used for its main indication in adults” (31). DDD enables comparison across different antipsychotic types and dosages and has been shown to align well with chlorpromazine equivalents in schizophrenia research (32,33). The participants were asked about their psychotropic drug use the past seven days and when possible, this was verified by checking their medical records. Serum concentrations of the three study drugs were available and, on average, remained within the therapeutic range throughout the study. The DDD numbers reported in this study indicate mean total daily antipsychotic use in the week prior to assessment and will be referred to in the discussion as total antipsychotic use (Sum DDD). Patients with no prior history of antipsychotic treatment at baseline, as indicated in their medical records, were classified as antipsychotic-naïve (AP-naïve).

Dopaminergic mechanism classification: To investigate potential differences related to pharmacodynamic mechanism, medications were categorized as dopamine antagonists (DAs) or dopamine partial agonists (DPAs). Aripiprazole was solely classified as a dopamine partial agonist (DPA), as no participants used brexpiprazole or cariprazine during the study. The following dopamine antagonists (DAs) in addition to amisulpride and olanzapine were registered in use by one or more participants: asenapine, chlorprothixene, clozapine, flupentixol, haloperidol, levomepromazine, paliperidone, prothipendyl, quetiapine, risperidone, zuclopenthixol and ziprasidone. Each participant was classified according to the antipsychotic(s) they used during the week prior to each assessment irrespective of original treatment allocation. Thus, participants who discontinued or switched from the original study medication continued to contribute data based on their current antipsychotic treatment. In cases of combined DPA and DA use, participants were classified as DPA users if the DPA dose exceeded a DDD of 0.2.

The neurocognitive assessment comprised of the following tests: D-KEFS FAS Verbal Fluency test (Delis et al., 2001), the Halsted-Reitan Trail Making test A and B (Reitan, 1992), The Hopkins Verbal Learning Test (HVLT –R) (Benedict et al., 1998), WAIS -IV Letter-number Span (Wechsler, 2008) and BACS Symbol coding (Keefe et al., 2004). For the HVLT-R we used alternate version at each test point. Together, these tests assessed key neurocognitive domains including processing speed, attention, executive functioning (working memory and cognitive flexibility), language-based verbal fluency, and verbal learning and episodic memory. Raw scores were transformed to t-scores using the scoring manual respective to each test. We report on the mean t- score of these tests, based on the number of tests that the participant completed.

### 2.4 Statistics

Descriptive statistics was performed in SPSS version 29 (IBM, 2022). To use all available data, the multivariate regression analyses were performed in MPlus version 8.11 (Muthén and Muthén, 2024).

We conducted a series of multiple regression models. Based on findings from previous research and the aim of achieving a parsimonious model, we employed a hierarchical regression approach using a three-step procedure. In the first step (Model 1), we included mPANSS level, cumulative antipsychotic exposure (Sum DDD), antipsychotic-naïve status, and dopamine partial agonist (DPA) as independent variables. This model served as the baseline estimated model, with all regression coefficients freely estimated without constraints. In the second step (Model 2), cognition was added to the model, given inconsistent findings in previous literature regarding its association with the outcome. In the third step (Model 3), interaction terms between Sum DDD and the other independent variables were included in order to examine moderation effects of cumulative antipsychotic exposure.

After the hierarchical regression analyses, a series of constrained models were estimated to examine whether predictor-outcome relations could be adequately described by an underlying linear trend across time (Newsom, 2015). This could only be done in the model that had the same independent variables at all timepoints, in Model 1. Time was coded based on assessment week and specified as the metric for the intercept and slope factors in the constrained latent growth curve models. First, all predictor-outcome relations were examined by intercepts and, where applicable, linear slopes. Second, non-significant slope factors were removed to achieve a parsimonious model, while corresponding intercept constraints were retained to preserve common relations over time. The final model retained intercept and slope constraints for mPANSS only.

Traditional fit indices are not meaningful in standard multivariate regression models as we estimate no structural relations between each timepoint. Explained variance was used as a predictive adequacy indicator in the unconstrained models (Kline, 2011). The fit of the final constrained model was evaluated relative to the freely estimated baseline model (Model 1) using −2 log likelihood-based (−2LL) model comparison (Newsom, 2015). This comparison assessed whether imposing level and linear trend constraints led to a statistically significant loss of model fit.

We tested for multicollinearity among the independent variables, and none of the main effect models yielded a tolerance value below 0.1 (Field, 2024). All continuous independent variables were centred to reduce multicollinearity in the interaction models. Statistical significance level was set at α = 0.05, two-tailed. We employed the maximum likelihood robust (MLR) estimator to utilise all available data under the full information maximum likelihood (FIML) framework and to account for potential skewness and kurtosis (Kline, 2011). Although the PANSS uses a 1-to-7 rating scale, it was treated as a continuous variable, consistent with standard practice, due to the relatively high number of response categories, moderate skewness (ranging from 0.58 to 1.02), and the use of the MLR estimator, which adjusts for non-normality (Norman, 2010). To reduce variance differences between mPANSS and cognition variables, we applied linear scaling by dividing them by constants (29 and 10, respectively). For mPANSS, 29 was chosen because it represents the number of items contributing to the total score, making the transformed value interpretable as an average per item. For cognition, 10 was selected pragmatically to achieve a similar scale and improve model estimation. Any positive constant would reduce variance without altering relationships between variables; these choices were made for interpretability and computational stability (Kline, 2011).

No formal correction for multiple testing was applied. The analyses were exploratory and aimed to characterise patterns of association within a limited set of theoretically related parameters derived from prespecified models, rather than to test a large number of independent, post-hoc hypotheses (Hooper, 2025). Applying strict multiplicity adjustments in this context would substantially reduce power and increase the risk of Type II errors, potentially obscuring meaningful signals in early-stage research. To mitigate the risk of inflated Type I error, analyses were organised into conceptually defined models and substantive conclusions were drawn only when associations showed consistent patterns across related model specifications. All results should therefore be regarded as hypothesis-generating and interpreted cautiously until replicated in confirmatory studies with prespecified hypotheses and formal error control.

Missing data were handled using full information maximum likelihood (FIML) under the assumption of missing at random (MAR), allowing efficient use of available data. The extent and patterns of missingness are reported in Table 2.

### 2.5 Ethics

The regional ethics committee in Norway, the Norwegian Medicines Agency, the Etikkommission der Medizinsche Universität Innsbruck and the Austrian Federal Office for Safety in Health Care (BASG) in Austria approved the study (ClinicalTrials.gov identifier: NCT01446328). The study adhered to the principles outlined in the Declaration of Helsinki and complied with the International Council for Harmonisation (ICH) guidelines for good clinical practice. Informed written consent was obtained from all participants prior to the baseline assessment. Travel expenses were reimbursed at each follow-up visit.

## 3 Results

### 3.1 Descriptives

A total of 144 participants were included in the study. Table 1 presents baseline characteristics of the sample. The mean insight level (PANSS G12) indicated minor impairment, ranging from 1 to 6 across the first four assessment points, and from 1 to 4 at the final point. Detailed descriptions of mean insight levels and other variables included in the analyses at each assessment point are provided in Table 2. The sample sizes reported in Table 2 represent all participants with available data at each assessment point, irrespective of subsequent medication changes. After the six-week assessment, insight scores declined across subsequent cross-sectional assessments, from 2.5 to 2.1, indicating improved insight. Cognitive functioning improved steadily over time, although it remained within the below-average range. The greatest symptom improvement, reflected by the lowest mPANSS score, was observed at the 12-month assessment. The proportion of participants receiving DPA treatment ranged from 21% to 29%. The number of antipsychotic-naïve participants was lowest at the 12-month time point.

**Table 1:** Descriptive Characteristics of the BeStInTro Sample (*N* = 144).

Variable	Value
Age (years), M (SD)	31.7 (12.7)
Female (%)	35.4%
Years of education, M (SD)	12.4 (3.0)
Antipsychotic-naïve (%)	39%
PANSS G12 Insight, M (SD)	3.42 (1.2)
mPANSS Total, M (SD)	75.0 (15.5)
PANSS Positive, M (SD)	21.2 (4.7)
PANSS Negative, M (SD)	17.83 (6.1)
PANSS General (excluding G12), M (SD)	36.0 (8.4)
Global Cognitive Performance (t-score), M (SD)	41.6 (8.3)
Defined Daily Dose (DDD), M (SD)	0.52 (0.84)
Partial Dopamine Agonist (%)	14.5%

**Notes:** M = Mean; SD = Standard Deviation. PANSS = Positive and Negative Syndrome Scale. DDD = Defined Daily Dose.

**Table 2:** Mean level of insight and variables of interest at each assessment point.

	6 weeks (*N* = 101)		12 weeks (*N* = 87)		6 months (*N* = 69)		12 months (*N* = 62)	
	*mean*	*SD*	*Mean*	*SD*	*Mean*	*SD*	*Mean*	*SD*
Insight G12	2.5	1.3	2.4	1.3	2.1	1.3	2.1	1.1
mPANSS	57.7	17.3	55.5	14.3	54.3	17.4	50.3	15.6
DDD	1.3	0.7	1.3	0.7	1.3	0.7	1.0	0.7
Cognition	43.3	7.6	N/A	N/A	46.3	7.6	47.2	8.7

	*%*	*n*	*%*	*n*	*%*	*n*	*%*	*n*
PDA	26.9	29/108	20.7	18/87	21.0	13/62	29.2	14/48
AP naïve	30.7	31	35.6	31	39.1	27	35.5	22

G12: General item number 12 in the Positive and Negative Syndrome Scale.

mPANSS: The Positive and Negative Syndrome Scale total score minus the insight item G12.

DDD: Defined Daily Dosage of antipsychotics.

Cognition: Global sum t-score.

PDA: Partial dopamine agonist, % of those using antipsychotics.

AP naïve: Participants not previously treated with antipsychotics before enrolled in the study.

SD: Standard Deviation.

N/A: not applicable, cognition was not assessed at 12 weeks.

### 3.2 Multivariable associations with the outcome

Table 3 shows how insight relates to each predictor and how much variance the models explain at different time points. Symptom severity (mPANSS) was the strongest and most consistent predictor: lower psychotic symptoms were linked to better illness awareness across almost all models and time points, except at six months in the interaction analysis. Interactions between symptom severity and antipsychotic dose (Sum DDD) appeared at week 6 and six months, where patients with severe symptoms on higher doses tended to have poorer insight.

**Table 3:** Variables associated with estimated insight level (PANSS item G12) at each assessment point.

	Baseline			6 weeks			12 weeks			6 months			12 months		
	*b*	*β*	*p*	*b*	*β*	*p*	*b*	*β*	*p*	*b*	*β*	*p*	*b*	*β*	*p*
**Model 1**															
mPANSS	0.60	0.27	**<0.001**	0.81	0.38	**<0.001**	0.62	0.24	**0.010**	1.11	0.51	**<0.001**	1.17	0.55	**<0.001**
PDA	−0.46	−0.13	0.306	0.15	0.05	0.573	−0.58	−0.18	0.114	−0.18	−0.06	0.619	0.16	0.07	0.690
Sum DDD	0.16	0.11	0.391	0.16	0.09	0.444	0.18	0.10	0.527	−0.29	−0.17	0.171	0.11	0.07	0.597
Naïve	0.37	0.16	0.199	−0.02	−0.01	0.954	0.17	0.07	0.588	0.48	0.19	0.098	0.48	0.02	0.880
R square	0.095			0.175			0.114			0.265			0.343		

**Model 2**															
mPANSS	0.45	0.20	**0.005**	0.82	0.39	**<0.001**	0.62	0.24	**0.011**	1.17	0.52	**<0.001**	1.17	0.55	**<0.001**
PDA	−0.52	−0.15	0.217	0.19	0.07	0.481	−0.57	−0.18	0.117	−0.19	−0.06	0.658	0.18	0.07	0.658
Sum DDD	0.12	0.08	0.515	0.17	0.09	0.432	0.18	0.10	0.518	−0.29	−0.17	0.522	0.15	0.09	0.522
Naïve	0.30	0.13	0.286	−0.02	−0.01	0.926	0.17	0.07	0.581	0.49	0.19	0.806	0.06	0.03	0.806
Cognition	−0.32	−0.22	**0.032**	−0.04	0.03	0.791	N/A	N/A	N/A	0.03	0.02	0.661	0.07	0.05	0.661
R square	0.139			0.177			0.113			0.270			0.345		

**Model 3**															
mPANSS	0.56	0.25	**0.002**	1.127	0.49	**<0.001**	0.59	0.22	**0.013**	0.33	0.15	0.481	0.92	0.41	**<0.001**
PDA	−1.04	-0.33	**0.040**	−0.18	−0.06	0.417	−0.28	−0.09	0.194	−1.08	−0.34	0.213	0.35	0.14	0.210
Sum DDD	−0.08	−0.06	0.639	−0.02	−0.01	0.928	0.19	0.10	0.449	−0.15	−0.09	0.473	−0.47	−0.24	0.061
Naïve	−0.85	0.34	0.404	0.35	0.12	0.135	0.34	0.12	0.248	0.64	0.23	**0.050**	0.15	0.06	0.472
Cognition	−0.23	−0.16	0.153	−0.02	−0.10	0.917	N/A	N/A	N/A	−0.45	−0.03	0.857	0.08	0.06	0.516
Sum DDD x mPANSS (1)	−0.28	−0.14	0.069	0.92	0.24	**<0.001**	0.31	0.07	0.313	0.41	0.35	0.103	−0.73	−0.21	**0.041**
Sum DDD x PDA (2)	0.78	0.35	**0.023**	−0.33	−0.08	0.334	−0.68	−0.17	**0.045**	0.90	0.39	0.156	1.70	0.51	**<0.001**
Sum DDD x Naïve (3)	2.09	0.44	0.391	0.85	0.25	**0.025**	0.60	0.27	0.058	-0.15	-0.04	0.803	0.65	0.18	0.175
Sum DDD x Cognition (4)	0.00	0.00	0.995	−0.61	−0.20	**0.030**	N/A	N/A	N/A	0.58	0.20	0.069	−0.60	−0.27	**0.007**
R square	0.160			0.319			0.167			0.377			0.507		

mPANSS: The Positive and Negative Syndrome Scale total score minus the insight item G12.

PDA: Partial dopamine agonist.

DDD: Defined Daily Dosage of antipsychotics.

Cognition: Global sum score.

b: Unstandardised regression coefficient.

β: Standardised regression coefficient.

p: *P*-value of unstandardised regression coefficient.

X: multiple / times.

N/A: not applicable, cognition was not assessed at 12 weeks.

R square: Explained variance of the model.

The other independent variables had more time-specific effects. Antipsychotic mechanism played a role at baseline: people who had used aripiprazole before the study showed better insight. This mechanism also interacted with dose at baseline, 12 weeks, and 12 months, but the strength and direction of these effects changed over time. Cognitive performance was linked to better insight at baseline and interacted with dose at week 6 and 12 months. Being antipsychotic-naïve at baseline predicted better insight at six months, but only in the interaction model.

**Fig. 1:**
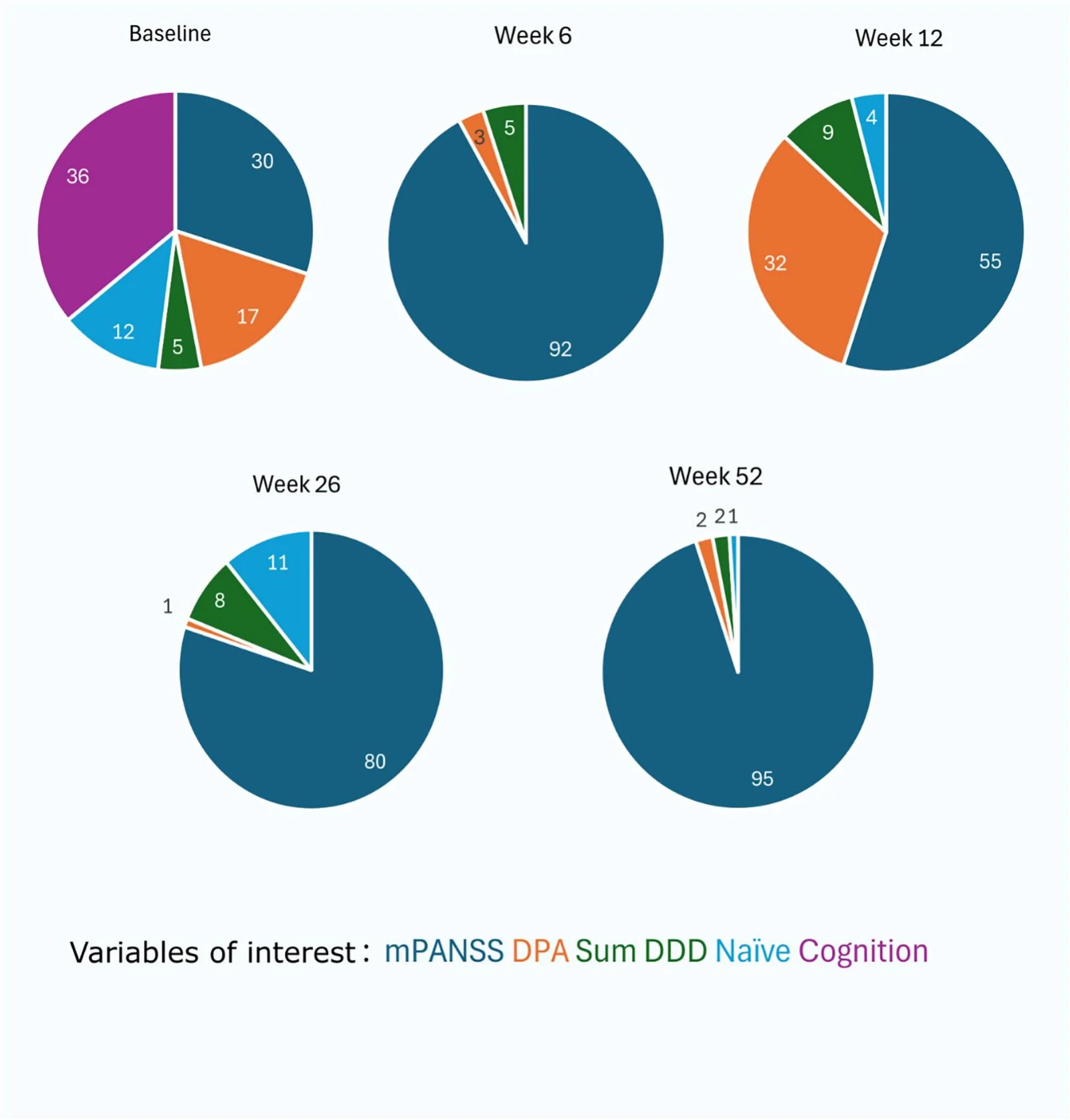
Percentage of explained variance from Model 2 at each time point.

Overall, models with interaction terms at 12 months explained the most variance. Fig. 1 shows how each predictor contributed to explained variance in Model 2 across all time points.

### 3.3 Level and change in relations

**Fig. 2:**
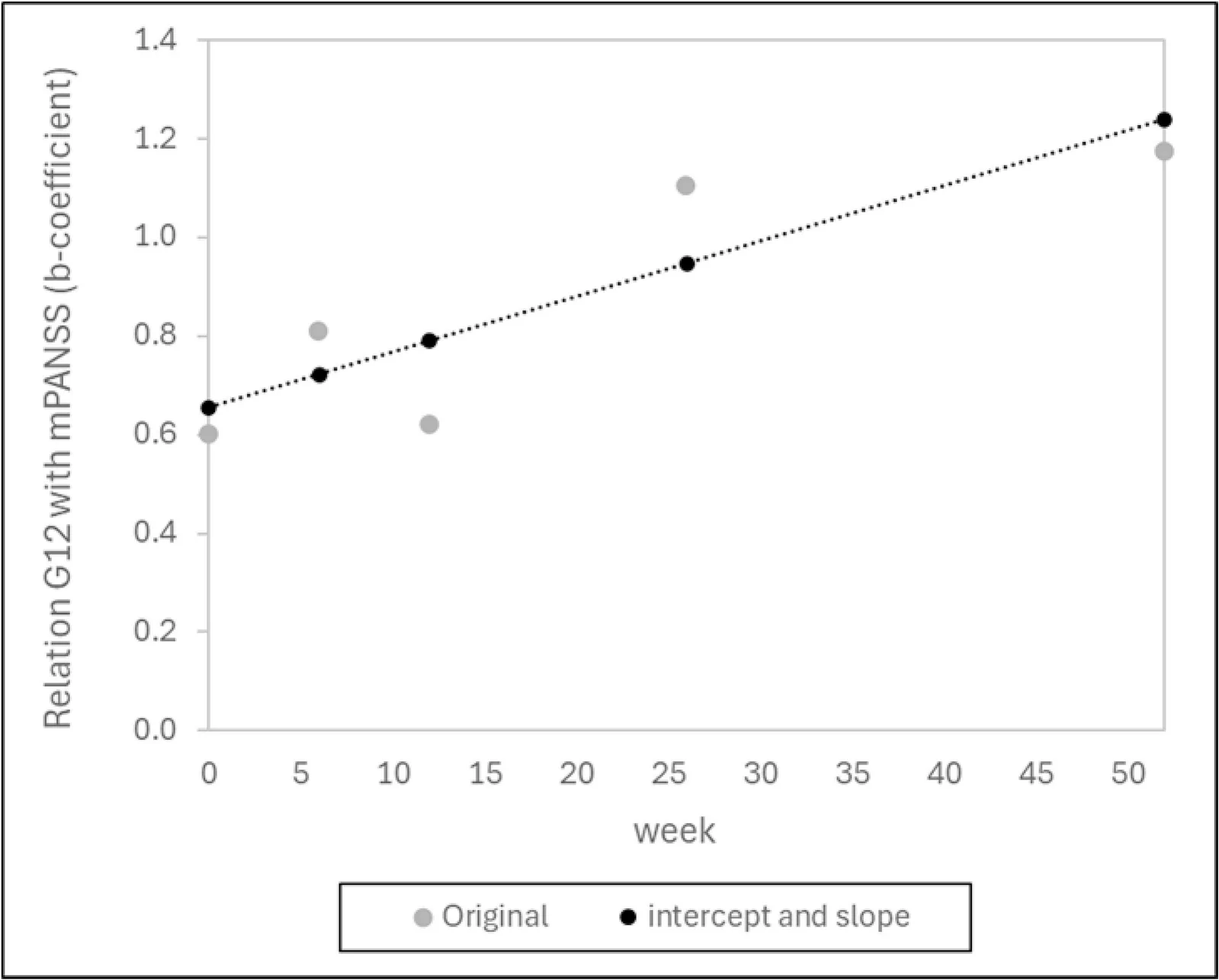
Regression coefficients between mPANSS and G12 over time.

The relationship between mPANSS and clinical insight (G12) was the only significant linear trend over time (interercept: α_0_ = 0.66, *p* < .001; slope: α_1_ = 0.01, *p* = .021). The regression coefficients for mPANSS were comparable to those observed in the freely estimated Model 1 and were all statistically significant at the *p* < .001 level. The estimated coefficients at baseline, 6 weeks, 12 weeks, 6 months, and 12 months were: b_w0_ = 0.66, β = 0.29; b_w6_ = 0.72, β = 0.34; b_w12_ = 0.79, β = 0.30; b_w26_ = 0.95, β = 0.45 and b_w52_ = 1.24, β = 0.57, respectively. The MLR-corrected -2LL difference test indicated no statistically significant difference in model fit between the free and the constrained models, χ^2^ (15) = 13.32, *p* = .57, indicating that the constrained model did not provide a statistically worse fit than the freely estimated model. The final results are shown in Fig. 2. Grey points represent freely estimated regression coefficients at each time point; black points represent coefficients implied by the longitudinal constrained intercept-and-slope model.

## 4 Discussion

Symptom severity consistently emerged as a predictor of lower insight levels. Total antipsychotic use was associated with insight only through its interaction with other independent variables; however, none of these effects were consistent across all time points. Findings related to patients treated with the partial dopamine antagonist (DPA) aripiprazole were mixed. Cognitive functioning was positively associated with insight at baseline and, in interaction with lower doses of antipsychotics at week 6 and at12 months. No consistent associations were found among the antipsychotic-naïve participants, although the few statistically significant findings indicated poorer insight.

At baseline, our sample showed a similar level of insight impairment to the dose reduction study (Gerretsen et al., 2017), with scores for the PANSS G12 item Lack of insight of 3.4 and 3.3, respectively. However, insight was less impaired in the CATIE sample, reporting a PANSS G12 score of 2.67 (Ozzoude et al., 2019). Symptom burden differed more markedly: our sample had higher mPANSS scores compared to the CATIE sample, reflecting a more active phase of psychosis. Baseline cognitive scores indicated low-average performance, comparable to the dose-reduction study, which reported a t-score of approximately 44.1. In contrast, the CATIE sample exhibited near-average cognitive functioning, with a z-score of −0.07, corresponding to a t-score of 49.3. Antipsychotic exposure was not directly comparable due to methodological differences between our study and the two former studies.

The mPANSS score was the only consistent predictor of insight across all five time points. In contrast, in the dose-reduction study symptom severity did not function as a stable predictor of insight, with no baseline association observed and a relationship emerging only at the 28-week post-reduction assessment (Gerretsen et al., 2017). Consistent with our findings, the CATIE study reported that greater illness severity predicted poorer insight at both baseline and the average of months 1 and 3 (Ozzoude et al., 2019). Collectively, these findings support a robust association between symptom severity and insight. Higher symptom severity has consistently been associated with poorer insight, as demonstrated in a systematic review (Lysaker et al., 2018). However, our results suggest that symptom severity may predict insight even at relatively low levels of clinical burden, indicating that this relationship is not confined to periods of active psychosis. Importantly, the association between mPANSS and insight was not only consistent across time points but also increased systematically in strength over time. Formal testing of time-varying effects indicated that this association could be adequately described by a linear trend. This suggests that the link between symptom burden and insight becomes more pronounced as patients progress through treatment and follow-up. These findings underscore the clinical importance of monitoring insight independently of symptom severity, particularly as their relationship appears to strengthen over time.

Cognition was an independent predictor of insight at baseline, consistent with findings from the dose reduction study (Gerretsen et al., 2017), whereas the CATIE study reported no significant association (Ozzoude et al., 2019). This variability across studies, possibly reflecting differences in sample characteristics and study design, suggests that cognition may not consistently predict insight independently. A systematic review supports this interpretation, reporting only weak associations between insight and cognitive subdomains (Subotnik et al., 2020). However, when examined in interaction with antipsychotic dosage, better cognitive functioning combined with lower doses was associated with greater insight, aligning with evidence that supratherapeutic dosing may impair cognitive performance (Sakurai et al., 2013). Our findings may point to a potential therapeutic window, where sufficient symptom reduction is achieved without compromising cognitive functioning and illness awareness. From a clinical perspective, this suggests that dose optimisation should not only aim for symptom control, but also consider preservation of cognitive functioning, particularly in patients where insight is a key treatment target. In such cases, careful dose adjustment may help balance these competing effects, although the observational nature of the data and the variability across time points warrant cautious interpretation.

Being antipsychotic-naïve predicted poorer insight at six months and through interaction with total antipsychotic use at six weeks. Using absence of prior treatment as a proxy for first-episode schizophrenia, these associations support evidence that first-episode patients typically show reduced insight at admission compared with multi-episode cases (Schennach et al., 2012). However, our findings emerged after sufficient time for treatment effects, suggesting impaired insight may persist beyond the acute phase in some first-episode patients. This aligns with findings showing that the illness phase moderates the relationship between insight, psychotic symptoms, and neurocognition. (Quee et al., 2014). At the same time, greater insight among multi-episode patients may partly reflect cumulative exposure to mental health services, psychoeducation, and previous treatment experiences, which could contribute to increased awareness and understanding of the illness.

Use of aripiprazole was associated with better insight at baseline and showed interaction effects with total antipsychotic dose across time. However, both the direction and strength of these associations varied, making the findings difficult to interpret. One possible explanation for these shifting patterns relates to the pharmacological profile of aripiprazole as a partial dopamine D₂ agonist. Unlike dopamine antagonists, aripiprazole may either reduce or enhance dopaminergic signalling depending on the prevailing level of endogenous dopamine activity, thereby contributing to dopamine system stabilisation (Cookson and Pimm, 2023; de Bartolomeis et al., 2015). These context-dependent effects may vary across stages of illness and may involve dopaminergic modulation within striatal circuits implicated in salience attribution and psychotic symptom formation (Kapur, 2003). Although no direct evidence from the present study can support this interpretation, one possible explanation is that the functional effects of aripiprazole vary according to the underlying dopaminergic state. In more acute phases, higher doses may be associated with reduced dopaminergic signalling, whereas during early stabilisation, partial D₂ agonism may facilitate a more optimal level of dopaminergic signalling and support improved insight. Over longer follow-up, adaptive changes or illness-related factors may again alter this relationship. These interpretations remain speculative but may help explain why the interaction between mechanism and dose changed over time. Overall, the relationship between dopamine D₂ receptor mechanisms and insight, both independently and in interaction with dosage, remained inconclusive. Thus, evidence that dopamine partial agonists provide superior outcomes with respect to insight or cognition remains inconsistent, and the clinical significance of distinguishing dopamine partial agonists from dopamine antagonists has not been established. Our findings therefore provide only limited support for a potential role of dopamine receptor mechanism in insight and should be interpreted as hypothesis-generating rather than confirmatory. While these findings do not exclude the impact of dopaminergic processes on clinical insight, they underscore the complexity of its underlying neurobiology. Larger studies are needed to clarify the nature and direction of these associations.

Although total antipsychotic use (Sum DDD) was not independently associated with insight, a statistically significant interaction involving Sum DDD and other variables associated with insight was observed. These findings suggest that the relationship between medication dose and insight is not uniform across patients. At week 6 and after 12 months, higher antipsychotic doses were associated with lower insight among individuals with more severe symptoms. In such cases, higher doses may reflect greater clinical burden, which itself is associated with poorer insight. These effects underscore the importance of considering patient history and symptom profile when evaluating the impact of pharmacological treatment on insight.

Our three statistical models demonstrated variable explanatory power, with R^2^ values ranging from 9.5% to over 50%. The highest explained variance occurred at 6 and 12 months, which may suggest that in more clinically stable samples, characterised by greater consistency in symptomatology and antipsychotic dosing, the independent variables included in the models exert a stronger influence on insight than during acute phases. Notably, the inclusion of interaction terms substantially increased explained variance across all time points which may underscore the importance of identifying optimal dosage in relation to other clinical variables. Furthermore, it reinforces the importance of moving beyond additive models to capture the nuanced interplay between clinical variables.

### 4.1 Strengths and limitations

This study adds several distinctions to the existing literature. We used DDD, which allowed us to include all antipsychotics. We also distinguished between DA and DPA treatments. Our sample was more diverse compared to previous studies, including not only individuals with multi-episode schizophrenia, but also those who were antipsychotic-naïve at baseline. While the repeated assessments enabled the examination of insight and its correlates across multiple stages of treatment, the current study deliberately focused on time-specific cross-sectional associations and therefore did not include longitudinal analyses of change. Given the repeated cross-sectional design, the directionality of the observed associations cannot be established. For example, greater symptom severity may contribute to poorer insight, whereas impaired insight may also adversely affect symptom course through reduced treatment engagement or other mechanisms. Future longitudinal analyses are needed to clarify these potentially bidirectional relationships. Nevertheless, the repeated assessment design provides a valuable foundation for future longitudinal studies aimed at clarifying the dynamic relationships between insight, psychopathology, cognition, and antipsychotic exposure.

This study has some limitations. Insight was assessed using a single item from the PANSS (item G12), which may not fully capture the multidimensional nature of the construct. Although PANSS item G12 has shown meaningful associations with more comprehensive insight measures (Sanz et al., 1998), it remains a global clinician-rated measure and may not capture all dimensions of insight and more comprehensive and reliable instruments might have produced different results (Pedhazur and Schmelldn, 1991). Similarly, using a patient-rated insight scale rather than a clinician-rated one could have yielded different outcomes, as self-assessments may reflect subjective awareness that is not always observable to clinicians. Furthermore, clinician-rated and self-rated measures may capture partly different aspects of insight. Self-rated measures have been suggested to reflect more stable, trait-related aspects of awareness, whereas clinician-rated measures may be more sensitive to changes in clinical state (Phahladira et al., 2019). Consequently, the associations observed in the present study should be interpreted as relating specifically to clinician-rated clinical insight rather than to insight as a broader multidimensional construct. Finally, the mean level of insight from six weeks onwards, which indicated potential impairment, might be difficult to detect clinically. While treatment allocation was based on a semi-randomised design, medication changes during the naturalistic follow-up were guided by clinical judgement, and residual confounding or selection effects therefore cannot be excluded. Grouping all dopamine antagonists into a single category may have oversimplified pharmacological differences, as these agents vary considerably in non—D₂ receptor profiles. This heterogeneity may have introduced variability that attenuated or obscured drug-specific effects. Furthermore, as aripiprazole was the only DPA used in our sample, findings cannot be generalised to the DPA class as a whole. As anticipated, the study also experienced considerable attrition over time. Nevertheless, all available data at each time point were utilised through the application of statistical software and the MLR estimator. However, attrition led to increased uncertainty in findings at later time points. Our preliminary results therefore require replication in larger studies. These limitations may have affected the sensitivity of the analyses and should be considered when interpreting the generalisability of the findings.

## 5 Conclusion

Symptom severity was the only variable consistently associated with clinical insight across all assessment points, and the strength of this association increased over time. These finding highlights the need to assess insight across the full symptom spectrum, including when symptoms are mild, and independently of antipsychotic dosage. No consistent evidence was found for differential effects on insight between dopamine antagonists and partial dopamine agonists. Future studies should further investigate the longitudinal interplay between symptom severity, antipsychotic dosage, and cognitive functioning to clarify their independent and interactive contributions to insight over time.

## CRediT authorship contribution statement

**Lena Antonsen Stabell:** Writing – original draft, Visualization, Investigation, Formal analysis, Data curation. **Erik Johnsen:** Writing – review & editing, Supervision, Investigation, Funding acquisition, Conceptualization. **Rune Andreas Kroken:** Writing – review & editing, Conceptualization. **Else-Marie Løberg:** Writing – review & editing, Conceptualization. **Inge Joa:** Writing – review & editing, Project administration. **Solveig Klæbo Reitan:** Writing – review & editing, Investigation. **Maria Rettenbacker:** Writing – review & editing, Investigation. **Rolf Gjestad:** Writing – review & editing, Supervision, Formal analysis.

## Declaration of competing interest

The authors declare that they have no known competing financial interests or personal relationships that could have appeared to influence the work reported in this paper.
